# Factors Affecting Daughter Cells' Arrangement during the Early Bacterial Divisions

**DOI:** 10.1371/journal.pone.0009147

**Published:** 2010-02-10

**Authors:** Pin-Tzu Su, Pei-Wen Yen, Shao-Hung Wang, Chi-Hung Lin, Arthur Chiou, Wan-Jr Syu

**Affiliations:** 1 Institute of Microbiology and Immunology, National Yang-Ming University, Taipei, Taiwan, Republic of China; 2 Institute of Biophotonics, National Yang-Ming University, Taipei, Taiwan, Republic of China; Tel Aviv University, Israel

## Abstract

On agar plates, daughter cells of *Escherichia coli* mutually slide and align side-by-side in parallel during the first round of binary fission. This phenomenon has been previously attributed to an elastic material that restricts apparently separated bacteria from being in string. We hypothesize that the interaction between bacteria and the underneath substratum may affect the arrangement of the daughter bacteria. To test this hypothesis, bacterial division on hyaluronic acid (HA) gel, as an alternative substratum, was examined. Consistent with our proposition, the HA gel differs from agar by suppressing the typical side-by-side alignments to a rare population. Examination of bacterial surface molecules that may contribute to the daughter cells' arrangement yielded an observation that, with disrupted *lpp*, the *E. coli* daughter cells increasingly formed non-typical patterns, i.e. neither sliding side-by-side in parallel nor forming elongated strings. Therefore, our results suggest strongly that the early cell patterning is affected by multiple interaction factors. With oscillatory optical tweezers, we further demonstrated that the interaction force decreased in bacteria without Lpp, a result substantiating our notion that the side-by-side sliding phenomenon directly reflects the strength of in-situ interaction between bacteria and substratum.

## Introduction

Bacterial biofilm formation and pathogenesis often involve an attachment of the bacteria to a specific surface to initiate the colonization. After such attachment, the bacterium is able to grow and replicate and finally this leads to population dominance. To observe the early events during bacterial number expansion, *E. coli* K-12 was grown on agar containing tryptone and yeast extract, and the formation of bacterial mini-colonies was studied by time-lapse video imaging [1]. It has been found that the daughter cells slide side-by-side during the first round of division and, after the second round of division, the resulting offspring cells are all arranged in 4-cell arrays. Although this observation could be traced back to much earlier reports [2], [3] and has been shown to be present in a variety of different settings [4]–[7], the driving forces that produce such an alignment remain obscure. It has been suggested that “an elastic material connecting apparently separated bacteria” [1], [8] is the cause of this phenomenon.

When viewing a bacterium lying on the surface of a horizontal substratum, several factors might affect the daughter cells' patterning while the bacterium is growing. (1) The bacterium encounters the gravitational force that is perpendicular to the substrate plane. (2) The cell grows and increases in volume along the bacterial poles, which will generate body elongation and result in an expansion force along the substrate surface. These two factors are universal to all bacteria whenever they are actively growing on a solid horizontal plane. (3) The elastic materials [1], [8], presumably tangled surface molecules, are expected to vary as the molecules involved change due to variation in the bacterial physiological states. However, because this “intrinsic” interaction would theoretically retain the daughter cells in their original positions, the force should be directionally acting against elongation. (4) Besides, these bacterial surface molecules may also interact with the substratum beneath the cells, in which diversely different ingredients may be involved. The latter interaction would constitute an “extrinsic” force that adds another restriction and acts against cell elongation. Finally, a motor force resulting from any rotary movement of flagella may disfavor ordered alignments and this disordering effect will also be aided by Brownian motion [7].

To evaluate the contribution of the extrinsic force(s) to bacterial patterning during early rounds of division, we varied substrata as well as employed bacteria containing a *fliG* deletion to examine the effect of the rotary motion of flagella on daughter cells' patterning. We observe that the classic 4-cell array is not always the preferred arrangement of the bacterial offspring after the second bacterial fission and the arrangement pattern may change if the substratum is changed. Moreover, we screened a variety of mutants including abortive syntheses of lipopolysaccharide (LPS), curli and outer membrane proteins to examine their effect on cell patterning after the first round of division. Among these mutants, we found that the offspring of *E. coli* lacking Lpp [9] decreased in 4-cell array population; besides, the interaction force between the bacterium and agar was smaller compared with the case of the other mutants. Moreover, Pal and TolB that form complexes with Lpp [10], [11] were also confirmed to have effects similar to Lpp on the daughter cells' patterning.

## Results

### Bacterial Cells' Patterning Affected by the Substratum

To characterize the patterning of bacterial cells during early divisions, *E. coli* K-12 BW25113 was grown on a Luria Bertani (LB) agar-coated slide with agar concentrations varied between 0.4% and 1.5% (w/v). Under the microscope equipped with time-lapse imaging, nearly all daughter bacteria were seen sliding side-by-side to each other right after the first fission, and then a 4-cell array was observed after the second division (data not shown). These observations show no apparent differences with those previously reported for another *E. coli* K-12 strain [1]. Daughter cell patterning observed with JW1923 (Table 1), a flagellum-null strain derived from BW25113 by disrupting *fliG*, indicated that the formation of the cell arrays on agar was not affected by the absence of flagella.

**Table 1:** Summary of daughter cells' patterns seen with different flagellum mutants^a^.

Strain	BW25113	JW1923	JW1879
Genotype	WT	Δ*fliG*	Δ*motA*
String^b^	0% (0)	0% (0)	0% (0)
Sliding^b^	95.2% (79)	93.0% (66)	97.8% (45)
Miscellaneous^b^	4.8% (4)	7.0% (5)	2.2% (1)
Total^b^	100% (83)	100% (71)	100% (46)

a Strains with the specified gene disrupted were derived from BW25113 and examinations were performed on LB-1.5% agar-coated slides.

b Daughter cells' pattern in percentage of the population; number of scored events shown in parentheses.

**Figure 1:**
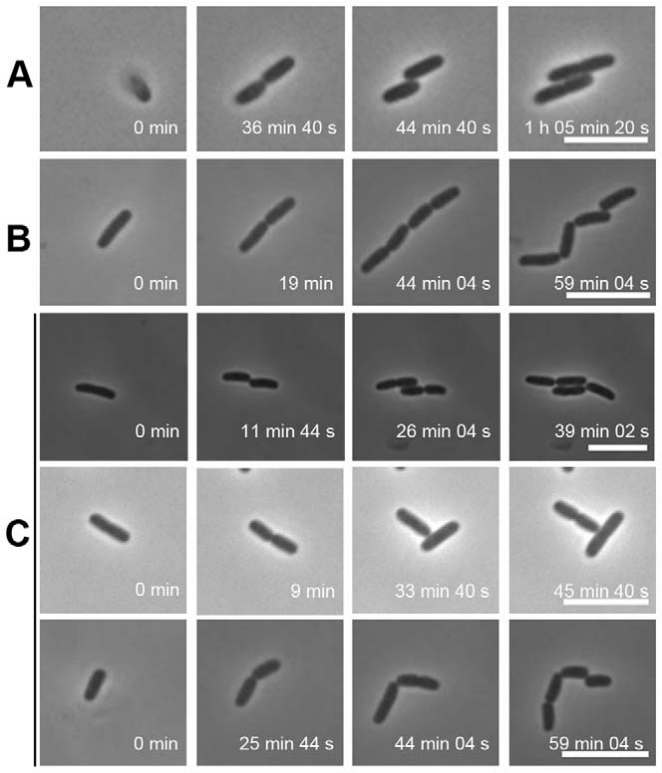
Appearance of daughter bacteria growing on LB-HA gel. JW1923, a *fliG*-deleted K-12 strain, was grown on a LB-HA (0.5%)-coated slide and the first two rounds of bacterial division were imaged by time-lapsed microscopy. (A) Formation of 4-cell arrays; (B) Patterning in a string; (C) Representatives of non-typical arrangements. Scale bar: 5 µm.

We reasoned that the interaction between the bacteria and the agar beneath the bacteria may play a critical role in the formation of the array structures. To examine this, the supporting agar matrix on slides was replaced with HA, a component of glycosaminoglycan with the ability to interfere with the adherence of microorganisms (US patent 6,428,903 B1) and perturb the attachment of *E. coli* to urothelium [12], [13]. Figure 1 shows the representative patterns of daughter cells taken from the flagellum-null JW1923 on the gel of LB-0.5% HA, including cells arrayed in parallel (2.7%; n = 110) (Figure 1A), linear strings of four cells (27.3%) (Figure 1B), and miscellaneous patterns (70.0%) (Figure 1C). To exclude the possible metabolism of bacteria with HA, cross-linked HA gel was used to repeat the experiments, and similar results were obtained. Interestingly, in comparison with JW1923, the parental BW25113 strain gave no cells at all forming 4-cell array under the same condition (data not shown). This observation suggests that the force generated by the flagellar motion may also affect the daughter cells' patterning.

**Figure 2:**
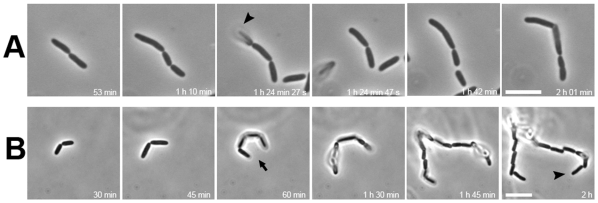
Patterning of daughter bacteria in liquid without underneath substratum. Bacterial divisions in LB medium were followed similar to that described in Figure 1. (A) Parental strain BW25113; (B) flagellum-null strain JW1923. In (A), arrowhead indicates a cell separating and disappearing from the bacterial string in the subsequent image. In (B), the arrow indicates bacteria that appeared as a string of four cells while the arrowhead marks a string of 16 offspring bacteria. Note: the balloon-type bacteria were not exactly on the focal plane of the microscope. No bacteria were observed to have a parallel or 4-cell array arrangement in all fields. Scale bar: 5 µm.

Overall, the observed percentage of bacteria arraying in parallel on the HA gel was notably smaller than that seen on a similarly prepared agar gel (<3% versus over 90%). This fact strongly supports the hypothesis that the components of the underneath substratum affects the early patterning of the daughter bacteria lying above. If this is the case, a complete elimination of the bacterium-substratum interaction ought to keep most of the offspring arranged in strings. To test this notion, we diluted the bacteria and restricted the bacteria to a thin LB-filled space sandwiched between two cover slips. Shown in Figure 2A is strain BW25113 that stayed in short strings in all fields. On tracing these early rounds of division using time-lapse imaging, bacteria were seen elongating and dividing into two in a string without any mutual sliding. Since flagellar rotary motion may accelerate string breakage of the daughter cells, the same experiment was repeated with strain JW1923 (flagellum null). This flagellum-null bacterium also gave strings of cells at various lengths. Figure 2B shows a developing string of 16 offspring remaining connected in the liquid. Population analysis, summarized in Table 2, shows that this flagellum-null JW1923 displayed more in long strings (≥3 cells, 16.5%) than that of the parental BW25113 strain (9.0%) in the same liquid culture condition. This observation strongly suggested that the flagellar rotation disfavors the linkage between divided bacterial cells. It is appropriate to re-emphasize at this point that no single pair of daughter bacteria was observed aligning side-by-side with either of the two strains in the liquid media.

**Table 2:** Effect of flagella on the string length of dividing bacteria in LB media.

Number in a string	BW25113^a^	JW1923 a
**1**	50.6% (378)	46.7% (430)
**2**	40.4% (302)	36.8% (339)
**3**	4.0% (30)	4.1% (38)
**4**	3.9% (29)	5.8% (53)
**5**	0.3% (2)	0.8% (7)
**6**	0.4% (3)	2.6% (24)
**7**	0% (0)	0.7% (6)
**8**	0.3% (2)	0.4% (4)
**>8**	0.1% (1)	2.1% (19)
**Subtotal for ≥3**	9.0% (67)	16.5% (151)
**Total**	100% (747)	100% (920)

a BW25113, the parental strain of flagellum-null JW1923; number of scored events shown in parentheses.

### Quantification of the Interaction Force between Bacterium and the Surrounding

**Figure 3:**
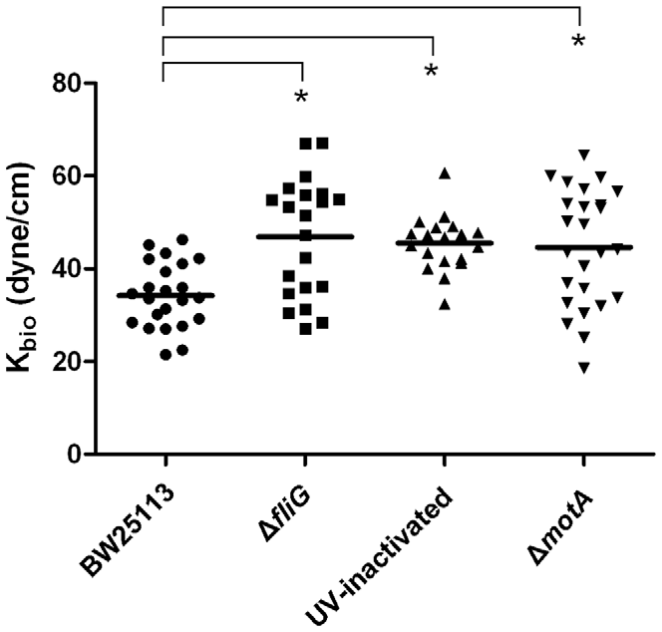
Measurement of the interaction of the bacterium with the surrounding LB agar in terms of the interaction force constant (K_bio_). The K_bio_ values, representing the interacting forces between bacterium and 0.2% LB-agar, were measured by oscillatory optical tweezers. In each group, about 20 measurements were performed and every single spot represents a measured K_bio_ value; the horizontal line marks the average of the group. Asterisk indicates that a statistically significant difference is observed between the paired strains (*p*<0.001 by *t*-test).

**Figure 4:**
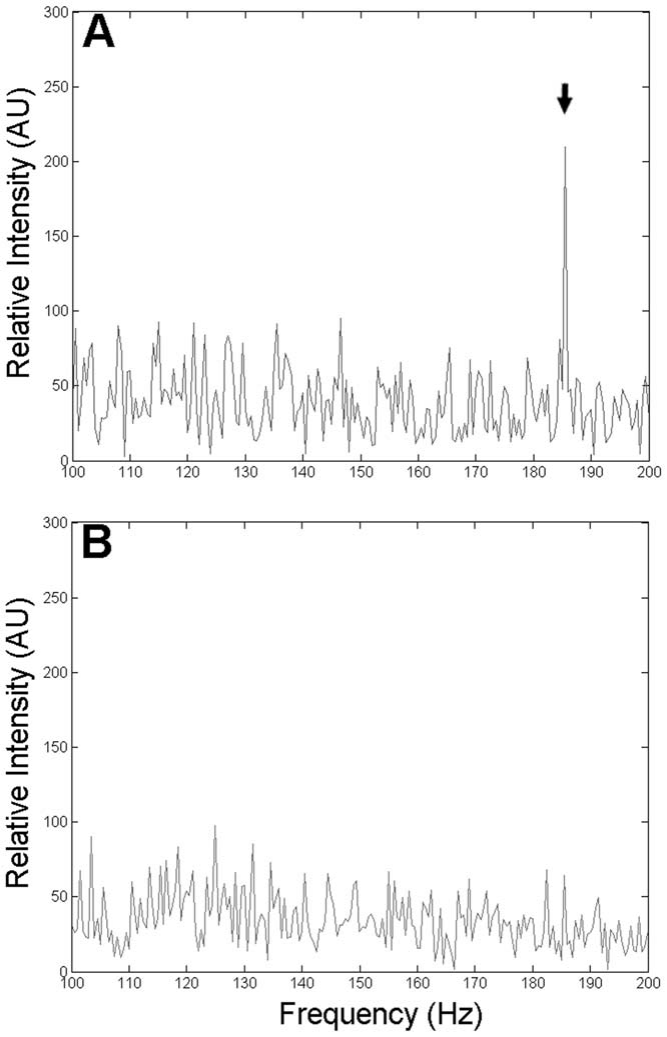
Comparison of flagellar rotation frequency. The flagellar rotation frequencies measured for strains BW25113 and JW1923 in LB medium were compared. A distinct peak was shown at around 185 Hz in the power spectrum of the quadrant photodiode signal for the case of the wild type strain BW25113 (A) but not in the flagella mutant strain JW1923 (B). Arrow indicates the position of the peak.

**Figure 5:**
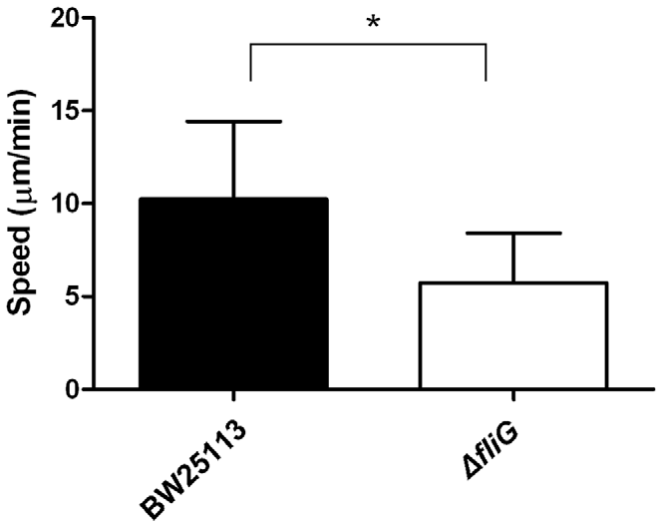
Measurement of bacterial motility. BW25113 and JW1923 in motility medium were tracked by time-lapsed microscopy for 10 min and the recorded images were analyzed to determine the average motility of each bacterium by MetaMorph software. The motilities of the two bacteria differed significantly at *P*<0.0001 (by *t*-test).

**Figure 6:**
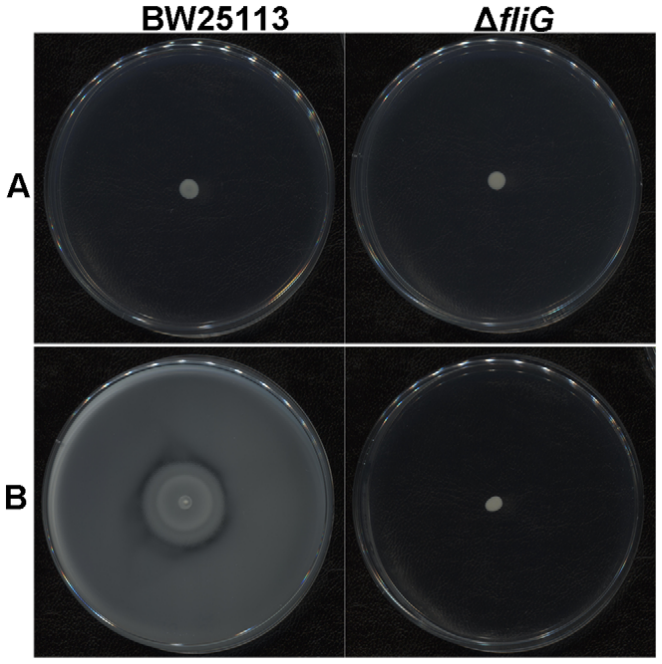
Bacterial motility detected on agar plates. Appropriately diluted bacteria were grown overnight at 37°C on LB plates containing 0.6% or 0.27% agar. No differences of bacterial colonies are seen in panel (A) (0.6% agar) whereas panel B (0.27% agar) shows that the parental BW25113 gave strong swarming activity that is not seen with the *ΔfliG* mutant.

To demonstrate that an interaction does exist between the bacterium and the substratum, the interaction force (represented by a force constant K_bio_) between a single bacterium and the surrounding agar gel (0.2%) in LB was measured by oscillatory optical tweezers (Figure 3); similar techniques have been applied successfully to measure the interaction between biomolecules and mammalian cells [14], [15]. The mean value of K_bio_ for strain BW25113 was 34.2±7.0 dyne/cm. The corresponding value for the *fliG*-deleted mutant JW1923 under the same condition was 46.9±12.7 dyne/cm. The increase in the interaction force constant in the absence of flagella implies that the interaction (between bacterium and 0.2% agar) is weakened probably by the rotary motion of the flagella. To confirm whether the mutation at *fliG* readily dampens the bacterial motility, strain BW25113 and the *fliG*-deleted mutant were trapped by optical tweezers, and the flagellar rotation frequencies were measured and compared. Besides, the motilities of these bacteria were analyzed by microscopic tracking. In the flagellar rotation frequency analysis, the power spectrum of BW25113 had a distinct peak at 185 Hz, which is absent in the spectrum of flagellum-null JW1923 (Figure 4). Consistent with this result, the moving speed of BW25113 in motility medium was significantly faster than that of JW1923 when analyzed in the travel-tracking experiments (Figure 5). In the agar motility assays, JW1923 apparently lost the ability of swarming in 0.27% agar that is inherited with the parental BW25113 strain (Figure 6).

To substantiate the subtle effect of flagellar motion on bacterial interaction with substratum, additional non-motile bacteria, i.e. UV-inactivated BW25113 and strain JW1879, of which *motA* coding for the flagellum-motor was disrupted [16], were examined for their interactions with 0.2% agar. The K_bio_ values of these bacteria were found to be similar to that of the *fliG* mutant (JW1923) and differed significantly from that of the parental BW25113 (Figure 3). These results further supported the above perception that the flagellar rotation perturbs and weakens the interaction between the bacterium and substratum.

### Effect of Lpp, Pal and TolB on Early Bacterial Cell Patterning

The above results demonstrated that interaction between bacterial surface and underneath substratum may vary in different settings and lead to different daughter cells' patterning. We next investigated whether there are additional factors on the bacterial surface other than flagellum that may also affect the early bacterial patterning. It is known that LPS and fimbriae on the surface of Gram-negative bacteria may serve as adhesive factors [17]–[19]. It has also been shown that outer membrane is involved in Gram-negative bacterial colonization [20]. Therefore, we examined whether daughter cells' alignment on LB-agar is affected by these bacterial factors. Strains with mutation at individual genes coding for proteins that either contribute to the syntheses of LPS and curli or directly function as outer membrane proteins or lipoproteins were examined. Among them, strain JW1667 with a disruption in gene *lpp* that encodes a membrane lipoprotein gave a remarkable difference. While the parental wild-type strain yielded 95.2% of daughter cells paired in parallel (n = 83), JW1667 gave only 29.7% (n = 74) under the same condition (Table 3, shown in bold). In contrast, other mutants with single genes defected at *wzzE*, which relaxes the regulation of the length of membrane O-antigen [21], *csgB*, which functions in curli nucleation [22], or *ompE*, which serves as a membrane porin, retained preferentially the pattern of daughter cells sliding in parallels (>80%) (Table 3). These observations suggest that the bacterial Lpp plays an important role that consequently affects the bacterial cells' patterning. To consolidate this notion, we investigated the Lpp-interacting proteins Pal and TolB [10], [11] by examining mutants JW0731 and JW5100 whose *pal* and *tolB* are disrupted, respectively. Consistent with the expectation, the percentage of cells sliding in parallel decreased with either the *pal* mutant (34.5%, n = 113) or the *tolB* mutant (40%, n = 70) (Table 3, shown in bold).

**Table 3:** Summary of daughter cells' patterns seen with various bacterial strains^a^.

Strain	BW25113^c^	JW1667	JW0731	JW5100	JW5601	JW1024	JW0231
Genotype	WT	Δ*lpp*	Δ*pal*	Δ*tolB*	Δ*wzzE*	Δ*csgB*	Δ*ompE*
String^b^	0% (0)	5.4% (4)	11.5% (13)	1.4% (1)	0% (0)	0% (0)	0% (0)
Sliding^b^	95.2% (79)	**29.7% (22)**	**34.5% (39)**	**40% (28)**	84.4% (124)	94.6% (87)	95.2% (79)
Miscellaneous^b^	4.8% (4)	64.9% (48)	54% (61)	58.6% (41)	15.6% (23)	5.4% (5)	4.8% (4)
Total^b^	100% (83)	100% (74)	100% (113)	100% (70)	100% (147)	100% (92)	100% (83)

a Strains with the specified gene disrupted were derived from BW25113 and examinations were performed on LB-1.5% agar-coated slides.

b Daughter cells' pattern in percentage of the population; number of scored events shown in parentheses.

c Numbers taken from Table 1 for easy comparison.

**Figure 7:**
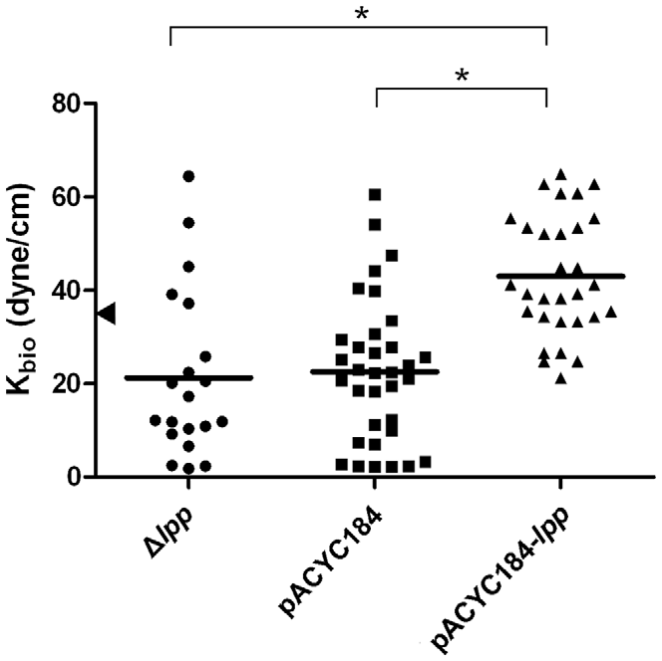
Interaction force constants measured for JW1667 and its related plasmid-transformed strains. JW1667 is an *lpp*-disrupted strain derived from BW25113 and has a phenotype with a low rate of daughter cells' patterning in parallel. K_bio_ measurement and illustrations of the measured values are as in Figure 3. Transformation of JW1667 with plasmid pACYC184-lpp was to complement the lost *lpp* whereas that with pACYC184 transformation was to give a plasmid control. The arrowhead marks the mean K_bio_ value of BW25113 shown in Figure 3. Asterisks mark the pairs with statistically significant differences (*p*<0.0001 by *t*-test).

To ensure that the perturbed daughter cells' patterning in strain JW1677 was readily due to the *lpp* disruption, the same experiments were repeated with a gene-complemented strain. JW1677 lost its tolerance to detergents and was broken up by 0.5% SDS solution. A proper complementation with exogenously expressed Lpp from plasmid pACYC184-lpp evidently restored JW1667 to its SDS resistance and the bacteria tolerated SDS to a high concentration (2%) as the parental BW25113 could withstand (Table 4). In contrast, when improperly complemented with a control plasmid, pACYC184, JW1667 remained as sensitive to SDS as it was. As to the side-by-side sliding pattern of daughter cells, Table 5 shows that pACYC184-lpp-transformed JW1667 behaved like the parental strain BW25113; in contrast, this did not happen to JW1667 transformed with pACYC184. Furthermore, the interaction forces with 0.2% agar gel of JW1667, pACYC184-lpp-transformed JW1667 and pACYC184-transformed JW1667 were measured with oscillatory optical tweezers in terms of K_bio_ and compared. Figure 7 shows that the mean K_bio_ value of JW1667 was lower than that of the parental strain BW25113. After transformation with pACYC184-lpp, the mean K_bio_ value was up-shifted and slightly higher than that of BW25113 whereas the same bacteria transformed with the control pACYC184 failed to do so. This result is consistent with the notion that the *lpp*-disrupted strain (i.e. JW1667) interacts weaker with agar, in comparison with the parental strain (i.e. BW25113), and this weakened interaction could be restored and perhaps strengthened by appropriate complementation with the Lpp expression.

**Table 4:** Bacterial resistance to detergent lysis.

Strains	SDS (%) resistance^a^
BW25113 (WT)	2
JW1667 (*lpp*-mutant)	<0.5
JW1667 transformed with pACYC184	<0.5
JW1667 transformed with pACYC184-lpp	2

a Expressed as the tested SDS concentration (w/v) in which bacteria behaved normally.

**Table 5:** Appropriate complementation converting the phenotype of JW1667 to that seen with the wild type (BW25113) in daughter cells' patterning.

Strain	BW25113^b^	JW1667	JW1667/pACYC184	JW1667/pACYC184-lpp
String^a^	0% (0)	5.4% (4)	4.7% (3)	0% (0)
Sliding^a^	**95.2% (79)**	29.7% (22)	26.5% (17)	**91.5% (65)**
Miscellaneous^a^	4.8% (4)	64.9% (48)	68.8% (44)	8.5% (6)
Total^a^	100% (83)	100% (74)	100% (64)	100% (71)

a Daughter cells' pattern in percentage of the population; number of scored events shown in parentheses.

b Numbers taken from Table 1 for easy comparison.

## Discussion

It is well recognized that bacteria act as multicellular organisms [8], [23], [24], [25] in establishing microbiota. Colonies grown on agar substrate may represent the simplest example of this type of interaction, which involves communication between bacteria of the same genetic background and their surroundings. Since a single bacterium could develop into a colony over a period of time by undergoing repeated fissions, the first two daughter cells at the beginning of colony formation may serve as a simplified model of the communications. Our observations on the patterning of daughter cells in different settings reflect the fact that such interactions do exist and may vary with the environmental conditions.

The surface structures of Gram-negative bacteria contain distinct molecular signatures but share common characteristics. In addition to bulk LPS on the surface, bacteria often bear many pili and perhaps a few flagella (from zero up to many), all of which protrude from the outer surface [26]. Furthermore, embedded in the LPS layer are many porins that act as gate channels for the transport of small molecules. Notwithstanding the complicated basic structure of the bacterium, the fact that each individual bacterium in a culture both metabolizes and increases bacterial mass continuously also needs to be taken into account. As a result, the bacterial body elongates along the long axis before fission, and the elongation generates an outward force against the surrounding. At the same time, gravity, flagellum's rotary action, Brownian motion and any tangled bacterial/substratum structure also affect this extending bacterium. Once the fission process is completed, the alignment and the relative position of the offspring were dictated by the summed up forces, a result leading to the specific arrangement of daughter cells seen in the microscope field.

In broth, the daughter cells form singlet or strings of different lengths. And in strain JW1923 without any flagellum, the string lengths in a population basis are apparently longer than those in the parental strain carrying flagella (Figure 2 and Table 2). This strongly supports the following notions. (1) A tip-to-tip interaction between two separated daughter cells does exist so that the cells are able to remain as a string in some circumstances. (2) The above interaction may be perturbed by Brownian motion and flagellar activity. The latter was supported by our observations that the longest case seen with the flagellum-positive BW25113 was a 9-cell string whereas the longest one seen with flagellum-null JW1923 was composed of 16 cells in a curling string (Table 2 and Figure 2B). (3) The hypothesis that the side-by-side alignment of two daughter cells results from an elastic material [1], [8] pulling back the two separated daughter cells is in conflict with the observation that such phenomenon does not exist during cell cultivation in liquid. However, the possibility that particularly sticky materials are sufficiently expressed only when bacteria make contact with agar surface could not be completely excluded.

As to why the early divisions of bacteria on agar give rise to a 4-cell array, our clues come from the fact that the side-by-side alignment of the daughter cells could be suppressed when growing on the gel of HA (Figure 1) or gelatin (data not shown). The influence of the substratum on the bacterial division pattern is most obvious when agar is used. Although it is difficult to precisely measure the force involved in the interaction between a bacterium and the agar surface, the fact that this interaction exists has been demonstrated by showing co-migration of a bacterium with an agar-coated bead that was manipulated by optical tweezers (data not shown). By trapping a bacterium suspended in the agar gel and oscillating it by oscillatory optical tweezers, the interaction force constant K_bio_ was measured. The K_bio_ values reflect the strength of the interaction between the optically trapped bacterium and the surrounding agar gel. Furthermore, an increase in the K_bio_ value in bacteria such as JW1923 (Δ*fliG*), JW1879 (Δ*motA*) and UV-inactivated wild type bacteria (Figure 3) strongly suggests that the flagellum motion may perturb the steady-state interactions between the bacterium and agar.

The initial attachment of a bacterium to a substrate may require numerous factors. Factors previously reported for adhesions include LPS, fimbriae, flagella, and outer membrane proteins [20], [27]. Based on these lines of information, we examined the daughter cells' patterning with a collection of *E. coli* strains with single mutation at genes linked to bacterial surface structures. These strains included mutations at *wzzE*, *csgA*, *csgB*, *csgC*, *csgD*, *csgE*, *csgF*, *csgG*, *fliG*, *motA*, *motB*, *ompA*, *ompC*, *ompE*, *ompF*, *ompG*, *ompN*, *ompR*, *ompT*, *ompW*, *ompX*, *lpp*, *pal*, *nlpB*, *tolB*, *mreB* and *ftsZ.* Unexpectedly, disrupting genes that are linked to LPS, curli or outer membrane proteins did not render bacteria to reduce 4-cell array patterning markedly. In contrast, the decreases in 4-cell array patterning of the Δ*lpp*, Δ*pal* and Δ*tolB* strains were so evident, in comparison with the parental wild-type strain, that the bacteria *per se* affect the daughter cells' patterning was strongly substantiated. These mutants grow at a normal growth rate but are more sensitive to detergent [11], [28]. While the exact role of Lpp is still under active investigation, it is currently known that Lpp is a major outer membrane lipoprotein [9] and has been reported to be crucial in maintaining the bacterial surface integrity [29]. If so, many structures on the surface of *E. coli* may delicately change in the absence of Lpp. This may explain why a single mutation hitting a single gene, such as *lpp*, of the bacteria perturbed the daughter cells' arrays considerably whereas abolishing a single structure, such as curli, on the bacterial surface hardly disrupted the adhesion of bacterium to cell surface [30].

In conclusion, our experiments shed light on a century-old mystery as to why *E. coli* cells are aligned in parallel during their earliest fissions. We suggest for the first time that the interactions between bacteria and the underlined substratum play an important role. We report the effect of substratum here and also emphasize that bacterial surface molecules/structures do participate in this interaction. This interaction force observed with agar is often strong enough to pull back the first round of divided cells along the rod-shape bacterial structure. In the case with HA gel, however, the strength of this force is often insufficient to fully counteract the expansion force so that the divided daughter cells rarely slide in parallel and often end up in a variety of patterns. This knowledge may lead us to reconsider how bacteria start to attach to different surfaces, gain an initial foothold when establishing a colony and ultimately to form a biofilm.

## Materials and Methods

### Bacterial Strains and Growth Conditions

*E. coli* strains used in this study are listed in Table 6. Bacteria were regularly cultured in LB broth at 37°C with continuous agitation. Kanamycin (50 µg/ml) was incorporated into media when mutants were used.

**Table 6:** *E. coli* strains used in this study.

Strain	Relevant genotype	Source
**JM109**	F' *traD36 proA^+^B^+^ lacI^q^ Δ (lacZ)M15/ Δ (lac-proAB) glnV44 e14^-^ gyrA96 recA1 relA1 endA1 thi hsdR17*)	New England Biolabs
**BW25113**	*rrnB3 ΔlacZ4787 hsdR514 Δ (araBAD)567 Δ (rhaBAD)568 rph-1*	The NARA Institute of Science and Technology
**JW1923**	*rrnB3 ΔlacZ4787 hsdR514 Δ (araBAD)567 Δ (rhaBAD)568 rph-1 fliG::kan*	The NARA Institute of Science and Technology
**JW1879**	*rrnB3 ΔlacZ4787 hsdR514 Δ (araBAD)567 Δ (rhaBAD)568 rph-1 motA::kan*	The NARA Institute of Science and Technology
**JW1667**	*rrnB3 ΔlacZ4787 hsdR514 Δ (araBAD)567 Δ (rhaBAD)568 rph-1 lpp::kan*	The NARA Institute of Science and Technology
**JW0731**	*rrnB3 ΔlacZ4787 hsdR514 Δ (araBAD)567 Δ (rhaBAD)568 rph-1 pal::kan*	The NARA Institute of Science and Technology
**JW5100**	*rrnB3 ΔlacZ4787 hsdR514 Δ (araBAD)567 Δ (rhaBAD)568 rph-1 tolB::kan*	The NARA Institute of Science and Technology
**JW5601**	*rrnB3 ΔlacZ4787 hsdR514 Δ (araBAD)567 Δ (rhaBAD)568 rph-1 wzzE::kan*	The NARA Institute of Science and Technology
**JW1024**	*rrnB3 ΔlacZ4787 hsdR514 Δ (araBAD)567 Δ (rhaBAD)568 rph-1 csgB::kan*	The NARA Institute of Science and Technology
**JW0231**	*rrnB3 ΔlacZ4787 hsdR514 Δ (araBAD)567 Δ (rhaBAD)568 rph-1 ompE::kan*	The NARA Institute of Science and Technology

### Observation of the Dividing Cells

Slides were coated with 100 µl LB containing molten agar at appropriate concentrations. Alternatively, HA, obtained from Sigma, was suspended in LB (0.5%; w/v) and boiled to dissolve. The solution was then applied to the slides (100 µl each). To prepare cross-linked HA, a method using N-hydroxysuccinimide and N-(3-dimethylaminopropyl)-N'-ethylcarbodiimide [31] was followed. All slides were freshly used after cooling down to room temperature.

Overnight cultured bacteria were 1∶100 diluted in LB. When the culture reached a density at OD_600_ between 0.3 and 0.6, they were appropriately diluted in LB, and 5 µl of the bacterial solution was transferred onto the gel-coated slide. After mounting a cover-glass, the slide was observed microscopically in a 37°C chamber with a Leica DM IRBE inverted microscope equipped with a 100×/1.40 N. A. oil objective (Leica 506042), an ORCA-ER digital camera (Hamamatsu C4742-95) and a peripheral controller (Hamamatsu C7418). Time-lapse images were taken every 20 sec in each channel using Aquacosmos software (Hamamatsu).

### Measurement of the Interaction Force Constant (K_bio_) by Oscillatory Optical Tweezers

To measure the interaction of the bacterium with the surrounding LB agar gel in terms of the interaction force constant K_bio_, a previously described method [15] was modified. In brief, when bacteria grew to an appropriate density, the bacterial solution was dropped into LB-agar to make a final agar concentration at 0.2% (w/v). 20 µl of the mixture were transferred into the sample chamber for oscillatory optical tweezers. The bacterium was trapped by a strongly focused (NA = 1.25, 100X) infra-red laser beam (optical power = 6 mW, wavelength = 1064 nm); with the focal spot oscillating with an amplitude of 32 nm and a constant oscillation frequency of 10 Hz, the interaction force constant (K_bio_) of the bacterium-agar interaction was determined by the following equation: 
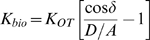



where “K_OT_”_,_ the spring constant of the optical trap, was pre-determined to be 9.9±0.3 pN/µm [32], [33]; “A”, the amplitude of the oscillatory trapping beam was 32 nm; “D”, the amplitude of the bacterium oscillation, and “δ”, the relative phase of bacterium oscillation relative to that of the beam focus, were measured by a quadrant photo diode in conjunction with a lock-in amplifier [15], [34].

### Measurement of the Flagellum Rotation Frequency

To measure the flagellar rotation frequency, the method previously described [35] was modified. In short, a bacterium was trapped by a pair of strongly focused (NA = 1.25, 100X) parallel laser beams (optical power = 12 mW, wavelength = 1064 nm and 633 nm) along the z-axis. The trapped bacterium with its long axis aligned along the x-axis between the foci of two laser beams was monitored under an inverted microscope equipped with a quadrant photodiode detector. The flagellar rotation frequency was obtained from the power spectrum of the quadrant photodiode output signal, representing the temporal variation along the y-axis in the xy plane.

### Measurement of Bacterial Motility

To measure the bacterial mobility, overnight-cultured bacteria were 1∶100 diluted in 3-ml fresh LB broth. When the culture density reached about OD_600_ at 0.5, the bacteria were spun down, washed, and re-suspended in appropriate amounts of motility medium (10 mM potassium phosphate at pH 7.0/0.1 mM EDTA/0.1 mM glucose/0.0002% (w/v) Tween 20) [35]. 200 µl of the bacterial suspension was transferred to a slide chamber, from which bacterium was monitored microscopically at 37°C under a Leica DM IRBE inverted microscope, equipped with a 100×/1.40 N. A. oil objective (Leica 506042), an ORCA-ER digital camera (Hamamatsu C4742-95) and a peripheral controller (Hamamatsu C7418). Time-lapse images were captured every second for 10 min using Aquacosmos software (Hamamatsu). The resulting images were used to compute the traveling distances of bacteria by MetaMorph program (Molecular Devices) and finally the bacterial motilities were calculated. To detect the bacterial motility with agar plates, 2 µl of overnight-cultured bacteria were spotted onto centers of individual LB-agar plates, of which agar concentration (w/v) was at either 0.6% or 0.27%. [36]. These plates were incubated at 37°C for 18 hr. Swarming motility of the bacteria was reflected by the enlarging diameters of colonies on the 0.27% LB agar plates when compared to the control.

### Plasmid Construction

To express Lpp, gene *lpp* along with a predicted promoter region was PCR amplified from the BW25113 chromosomal DNA with primers 5′-GGCCAAGCTTTTTTTTATTTAATCGATAACCAGAAGCAA-3′ and 5′-GGCCGGATCCTTACTTGCGGTATTTAGTAGCCATGTT-3′, in which sites recognized by restriction enzymes are underlined. The PCR product was digested with *Hin*dIII and *Bam*HI and subsequently cloned into pACYC184 previously digested with the same enzymes to generate pACYC184-lpp.

### Bacterial Sensitivity to Detergent SDS

*Salmonella* with *lpp* deleted has been reported with a phenotype of sensitivity toward SDS and the method described [37] to test this phenotype was followed. In brief, overnight-cultured bacteria were 50-fold diluted into a fresh medium and grown to OD_600_ about 0.4. A stock solution of SDS (10%; w/v) was added to the culture to a final SDS concentration at 0, 0.5, 1 and 2%. The cultures were then agitated at 37°C for 3 h. OD_600_ was measured before and after the agitation and a 50% or more reduction in optical density represents the sensitivity of the bacteria to the SDS concentration. Results were confirmed by three independent experiments.
